# A dual-functional electrolyte additive displaying hydrogen bond fusion enables highly reversible aqueous zinc ion batteries

**DOI:** 10.1038/s42004-024-01259-3

**Published:** 2024-08-08

**Authors:** Qiuxia Zhang, Xuan Gao, Kejiang Liu, Nan Gao, Shaoheng Cheng, Yuhang Dai, Haobo Dong, Junsong Liu, Guanjie He, Hongdong Li

**Affiliations:** 1 State Key Laboratory of Superhard Materials, College of Physics, Jilin University, Jilin, Changchun 130012 PR China; 2 Christopher Ingold Laboratory, Department of Chemistry, University College London, 20 Gordon Street, London, WC1H 0AJ UK; 3 Department of Engineering Science, University of Oxford, 17 Parks Road, Oxford, OX1 3PJ UK; 4 School of Future Technology, South China University of Technology, 381 Wushan Road, Tianhe District, Guangzhou, 510641 PR China

**Keywords:** Batteries, Energy

## Abstract

In recent years, aqueous zinc-ion batteries (AZIBs) have attracted significant attention in energy storage due to their notable advantages, including high safety, low cost, high capacity, and environmental friendliness. However, side reactions like hydrogen evolution and zinc (Zn) dendrites can significantly impact their Coulombic efficiency (CE) and lifespan. Effectively addressing these issues has become a focus of research in this field. In our study, dimethyl sulfoxide (DMSO) and nanodiamonds (NDs) were used to optimize the electrolyte of AZIBs. Benefiting from the hydrogen bond fusion of DMSO and NDs, which regulates the Zn deposition behavior, effectively inhibiting the growth of Zn dendrites, hydrogen evolution, and corrosion. The Zn | |Zn symmetric cells using NDs-DMSO-ZS demonstrate exceptional cycling stability for over 1500 h at 1 mA cm^−^^2^, while the Zn//Cu asymmetric cells achieve up to 99.8% CE at 2 mA cm^−^^2^. This study not only shows the application prospects of electrolyte optimization in enhancing AZIBs performance, but also provides a reference for the advancement of electrolyte technology in advanced AZIBs technology.

## Abstract (web summary)

Aqueous zinc ion batteries represent promising next-generation energy storage systems, but unwanted side reactions such as hydrogen evolution and zinc dendrite formation can significantly impact their Coulombic efficiency and lifespan. Here, dimethyl sulfoxide and nanodiamond additives are introduced to a ZnSO_4_ electrolyte, effectively inhibiting side reactions and dendrite formation through hydrogen bond fusion, and enabling exceptional cycling stability for over 1500 h at 1 mA cm^−^^2^.

## Introduction

With the continuous growth of the demand for renewable energy in contemporary society, there is a growing need to optimize the production and utilization methods. Consequently, the development of large-scale energy storage systems (ESSs) with high energy density, enhanced safety, and environmental friendliness has become imperative^1,2^. Among numerous solutions, aqueous zinc-ion batteries (AZIBs) stand out due to their higher theoretical capacity (820 mAh g^−1^), lower redox potential (−0.76 V vs. the standard hydrogen electrode (SHE)), and lower production costs (given the abundance of zinc (Zn) metal in the Earth’s crust at 0.075%)^3–6^. Due to the excellent prospects of AZIBs, many researchers have conducted in-depth research on them. With the continuous development of electrode, separator, and electrolyte materials, the performance of AZIBs continues to reach new highs, while also promoting the commercialization of AZIBs^7–11^. However, some issues like the growth of Zn dendrites, hydrogen evolution, and corrosion significantly impact the performance of the cells, primarily manifested by lower Zn anode utilization, reduced Coulombic efficiency (CE), and rapid capacity decay^12,13^. Therefore, it is necessary to improve the performance of the battery by optimizing its internal structure.

Specifically, during the Zn deposition process, Zn^2+^ needs to overcome the energy barrier to de-solvate and release water molecules from the Zn^2+^ solvation sheath before being reduced to form Zn. However, due to the strong Coulombic interactions between the solvated Zn^2+^ and its surrounding H_2_O shell, the potential will be increased during the Zn^2+^ de-solvation process. The elevated potential accelerates the decomposition of active free H_2_O, leading to side reactions such as hydrogen evolution and corrosion on the Zn anode surface^14–16^. Furthermore, the hydrogen evolution reactions (HER) can lead to an increase in the surrounding pH, promoting the formation of Zn^2+^ passivation layer (such as inactive by-products ${{Zn}}_{4}{({OH})}_{6}{{SO}}_{4}\cdot x{H}_{2}O$, ZHS) and the growth of Zn dendrites^17,18^. In addition, during the charging-discharging process, the Zn^2+^ plating/stripping occurs continuously at the electrode-electrolyte interface (EEI). Therefore, the kinetics of Zn^2+^ also seriously affect the uniform Zn deposition, as well as the reversibility and lifespan of the cells. From an electrochemical perspective, the kinetics is reflected through electrode polarization, which includes three aspects: concentration polarization, electrochemical polarization, and Ohmic polarization^19,20^. These unfavorable factors mentioned above do not exist alone, but are interrelated and interact with each other, which jointly affect the performance of AZIBs.

Among the strategies to inhibit the growth of Zn dendrites, hydrogen evolution, and corrosion, the regulation of Zn^2+^ solvation sheath is one of the relatively effective methods^21–24^. Wu et al. have published an influential study that involves dimethyl sulfoxide (DMSO) as a cost-effective electrolyte additive for AZIBs, specifically in the form of ZnSO_4_/H_2_O-DMSO electrolyte^15^. As an important organic solvent, DMSO has the characteristics of high polarity, high boiling point (189 曟 at 1 atm), constant (46.45), high donor number (29.8), and compatibility with water^14,25^. On the one hand, the strong interaction between H_2_O and DMSO decreases the quantity of active free H_2_O. On the other hand, DMSO can preferentially participate in the Zn^2+^ solvation sheath, reducing the amount of active free H_2_O at the electrode-electrolyte interface by reducing the amount of solvated H_2_O. In addition, the organic molecules have shorter chain lengths and smaller end steric hindrances, making them more likely to cover protruding parts and delay the Zn deposition kinetics. Using DMSO as an electrolyte additive can optimize the Zn deposition kinetics, and effectively inhibit the growth of Zn dendrites and other side reactions (hydrogen evolution, corrosion). However, too strong adsorption of organic molecules will cause serious electrode polarization, increasing the impedance at EEI to a certain degree^26,27^. In the past few decades, new nanotechnologies and materials have been confirmed to be used in energy related fields, including energy storage and conversion. The detonation nanodiamond particles (NDs) have the dual characteristics of diamond and nanomaterials, not only possessing the ultra-high hardness and good chemical stability of diamond, but also possessing the unique characteristics of nanomaterials, such as large specific surface area and small-size effect. NDs with unique advantages stand out among numerous carbon nanomaterials and have shown great potential in energy storage applications^28^. In addition, they have a large number of surface functional groups (such as hydroxyl (-OH), carboxylic (-COOH), and carbonyl (C = O)), which determine the chemical properties of NDs^29,30^. However, due to the action of van der Waals forces, they form tightly bound primary particle agglomerates during synthesis and purification, which severely limits their applications^31^. Fortunately, using DMSO as a solvent for NDs can improve the resistance to agglomeration and sedimentation in suspensions for NDs with positive zeta potential, which is beneficial for the de-aggregation of NDs^32^. More importantly, DMSO and NDs can be used as electrolyte additives to exert synergistic effects in optimizing the electrolyte. Specifically, the oxygen-containing functional groups (-OH, -COOH) of NDs and the S = O groups of DMSO can interact by forming hydrogen bonds (HBs). HB is a unique noncovalent bond that can positively affect the properties of electrodes and electrolytes, thereby improving the electrochemical performance of energy storage devices^33^. DMSO molecules can quickly detach from the [Zn(H_2_O)_m_DMSO_n_]^2+^ structure under the action of the HBs, and timely released Zn^2+^ to participate in the Zn^2+^ plating/stripping process. Improving reaction kinetics, namely reducing electrode polarization^34–36^.

In this work, we introduced an additive containing DMSO and NDs (NDs-DMSO), expected to demonstrate a synergistic effect in inhibiting the growth of Zn dendrites, hydrogen evolution, and corrosion. On the one hand, DMSO can optimize the Zn deposition kinetics. On the other hand, thanks to the HBs between the surface functional groups of NDs and DMSO molecules, the effective reduction of electrode polarization caused by DMSO is achieved through the HBs. The experimental results indicate that the Zn | |Zn symmetric cells using ZnSO_4_ electrolyte with NDs-DMSO (NDs-DMSO-ZS) exhibit a consistently stable charge-discharge profile compared to the control groups (ZnSO_4_ electrolyte with DMSO (DMSO-ZS) and the pure ZnSO_4_ electrolyte (ZS)), maintaining an exceptionally long cycling life of over 1500 h. The Zn | |Zn symmetric cells using NDs-DMSO-ZS also demonstrate outstanding cycling stability, exceeding 800 h at a current density of 3 mA cm^−^^2^. In addition, the Zn//Cu asymmetric cells exhibit a highly significant improvement in reversibility with an average CE (99.8%) over 800 cycles at 2 mA cm^−2^ and an area capacity of 1 mAh cm^−^^2^. This work provides a unique additive that effectively inhibits the growth of Zn dendrites and reduces various side reactions, providing a reliable idea for accelerating the commercial development of AZIBs.

## Results and Discussion

**Fig. 1:**
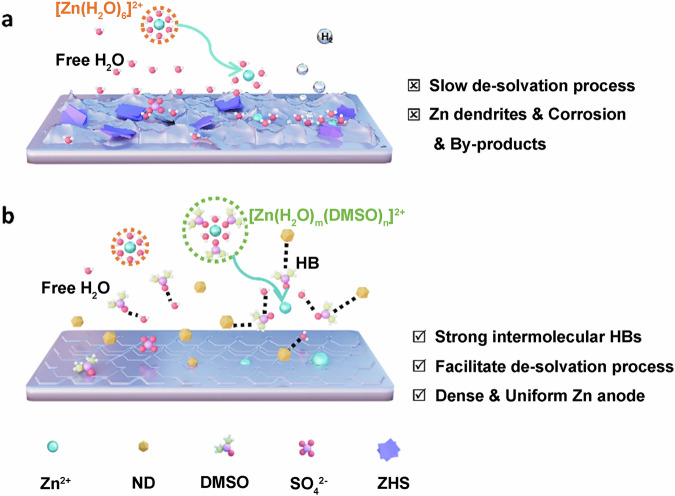
Schematics of the Zn^2+^ plating/stripping behaviors. **a** The Zn anode surface in ZS. **b** The Zn anode surface in NDs-DMSO-ZS.

The Zn dendrites and other side reactions (hydrogen evolution, corrosion) are closely related to EEI. Interfacial engineering, such as electrolyte additives, can effectively inhibit the Zn dendrites and other side reactions by regulating the Zn deposition behavior^37,38^. Specifically, during the Zn^2+^ plating/stripping process, as shown in Fig. 1a. Unfortunately, Zn^2+^ in the electrolyte often exists in the form of hydrated Zn^2+^, with each Zn^2+^ surrounded by six combining H_2_O. Therefore, the solvated [Zn(H_2_O)_6_]^2+^ must consume a certain amount of energy to de-solvate and release a large amount of solvated H_2_O to form Zn^2+^, and the released solvated H_2_O forms active free H_2_O. The H–O bonds of active free H_2_O are relatively weak, thus leading to the deprotonation of H_2_O, promoting the decomposition of active free H_2_O and producing H_2_. Subsequently, the H^+^ is continuously consumed, increasing the local pH value, which Zn^2+^ and [Zn(H_2_O)_6_]^2+^ on the Zn anode surface are more likely to participate in side reactions, forming low conductivity by-products (ZHS). These low conductivity by-products grow recklessly on the Zn anode surface, making the surface rough and changing the initial uniform distribution of current, triggering the growth of Zn dendrites^16,39^. In this situation, it is possible to regulate the structure of [Zn(H_2_O)_6_]^2+^, reduce the amount of active free H_2_O, and achieve a dendrite-free zinc ion battery with long-term electrochemical stability^40^.

Therefore, we attempted to introduce NDs-DMSO additive into 2 M ZnSO_4_ electrolyte. As shown in Fig. 1b, this additive plays a crucial role in inhibiting Zn dendrites and other side reactions. Due to the organic functional group (S = O), DMSO can act as a hydrogen bonding acceptor (HB acceptor) to form strong coordination interactions with H_2_O, reducing the amount of active free H_2_O^15^. In addition, DMSO can also participate in the [Zn(H_2_O)_6_]^2+^ structure and optimize the Zn^2+^ solvation sheath, which can regulate the Zn deposition behavior and inhibit the growth of Zn dendrites and other side reactions^17^. However, as a highly polar organic additive, DMSO has a strong adsorption capacity for Zn^2+^, leading to severe electrode polarization and cell failure^26^. NDs terminated by various oxygen-containing surface functional groups have shown enormous potential in energy storage applications. The surface chemical properties of NDs were characterized by FTIR, as shown in Fig. S1. Several strong absorption peaks at approximately 1089, 1630/1758, and 3433 cm^−^^1^, correspond to the stretching vibrations of C-O, COO^-^, and O-H bonds^29,41,42^. The FTIR results indicate the existence of many -OH and -COOH functional groups on the NDs’ surface. Due to the presence of surface functional groups like -OH, -COOH, and C = O, NDs can act as both hydrogen bonding donor (HB donor) and hydrogen bonding acceptor (HB acceptor), which NDs can form HBs with DMSO to better assist in Zn deposition and reduce electrode polarization^34,36^. Specifically, during the Zn^2+^ de-solvation process, the [Zn(H_2_O)_m_DMSO_n_]^2+^ must decouple and release DMSO and water molecules to form Zn^2+^. However, DMSO has a strong adsorption capacity for Zn^2+^, so the de-solvation process requires more energy consumption, resulting in slower reaction kinetics and increased electrode polarization. Fortunately, OH and -COOH on the surface of NDs as HB donor groups and the S = O groups of DMSO as HB acceptor groups to form the HBs, and DMSO molecules can quickly detach from the [Zn(H_2_O)_m_DMSO_n_]^2+^ structure under the action of HBs. The strong intermolecular HBs decrease unnecessary energy consumption during the de-solvation process, improve reaction kinetics, and reduce electrode polarization. As an electrolyte additive, NDs-DMSO optimizes the electrolyte, regulates the Zn deposition behavior, and achieves a dense and uniform Zn anode surface.

**Fig. 2:**
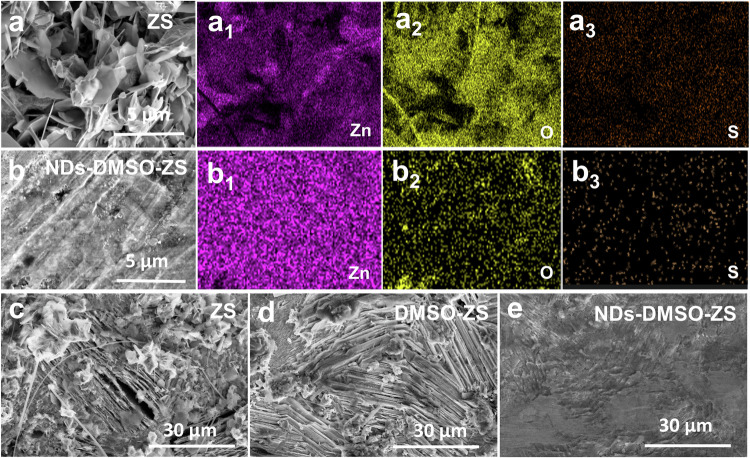
Morphology change of the Zn anode surface after 2 h cycling at 10 mA cm^−^^2^ for 10 mAh cm^−^^2^. **a, a**_**1**_–**a**_**3**_ SEM images and the corresponding EDS elemental maps of the Zn anode surface in ZS. **b, b**_**1**_–**b**_**3**_ SEM images and the corresponding EDS elemental maps of the Zn anode surface in NDs-DMSO-ZS. The SEM images of the Zn anode surface after 20 h cycling at 1 mA cm^−^^2^ for 1 mAh cm^−^^2^ in (**c**) ZS, (**d**) DMSO-ZS, and (**e**) NDs-DMSO-ZS.

Scanning electron microscopy (SEM) was performed to clarify the morphology evolution of the Zn anode surface in Zn | |Zn symmetric cells with different electrolytes and cycles. As shown in Fig. 2a, after cycling for 2 hours under a current density of 10 mA cm^−^^2^, the Zn anode surface using ZS is covered by numerous plate-like protrusions, which indicate uneven Zn deposition behavior. During the charging-discharging process, these plate-like Zn dendrites continuously grow along the separator. Eventually, the Zn dendrites may penetrate the separator to cause short-circuiting and separate from the Zn anode surface to form so-called “dead Zn”^15^. The corresponding energy-dispersive X-ray spectroscopy (EDS) results indicate a higher content of the O and S elements (Fig. 2a_1_–2a_3_, Fig. S2a), further confirming the formation of the by-products (ZHS). More importantly, the by-products of low conductivity usually form a passivation layer on the Zn anode surface, thereby reducing the Zn utilization and affecting the Zn deposition kinetics^43^. By contrast, the Zn anode surface using NDs-DMSO-ZS doesn’t exhibit particularly prominent plate-like Zn dendrites during the cycle process (Fig. 2b), suggesting relatively steady Zn deposition behavior. The EDS results also show that there is uniform Zn elements distribution and fewer by-products. (Fig. 2b_1_–2b_3_, Fig. S2b). Fig. S3 further investigates the ZHS of these different electrolytes using the X-ray diffraction (XRD) tests. During the discharge phase, the HER causes an increase in OH^-^ concentration, promoting the production of ZHS at EEI. During the charging phase, the disappearance of ZHS indicates good reversibility of the cathode materials^44–46^. For Mn‐based cathode material, the formation of ZHS in the above process can be summarized as follows^47^:

1 $$4{Zn}^{2+}+6{{OH}}^{-}+4{{SO}}_{4}^{2-}+x{H}_{2}O\leftrightarrow {{Zn}}_{4}{({OH})}_{6}{{SO}}_{4}\cdot x{H}_{2}O$$

At the first full discharge (0.8 V), as shown in Fig. S3a, ZHS was formed on the cathode of the three electrolytes, but the diffraction peak intensity of NDs-DMSO-ZS was the weakest, indicating a lower content of ZHS (approximately located at 8.2°). At the first full charge (1.9 V), as shown in Fig. S3b, only the diffraction peak intensity of NDs-DMSO-ZS significantly decreased. When subjected to the second discharge (1.6 V), as shown in Fig. S3c, the diffraction peak intensity of NDs-DMSO-ZS increased but remained the weakest. By comparing different potentials, the cathode surface of DMSO-ZS and ZS accumulated a large number of ZHS. The intensity change of the ZHS diffraction peak of NDs-DMSO-ZS indicates that it has good cycling stability. In addition, Zn | |Zn symmetric cells were assembled using three different electrolytes and tested under a current density of 1 mA cm^−2^. After cycling, the morphology of their Zn anodes was characterized by SEM, as shown in Fig. 2c, d, e. The SEM results show that after 10 cycles of the cells, the Zn anode surface using ZS is covered with disordered and irregular dendrites, while the DMSO-ZS is also covered with a small number of dendrites, indicating that uneven Zn deposition leads to the formation of dendrites, which seriously affects the electrochemical performance and ultimately makes the cell failure^48,49^. In contrast, the surface of the Zn anode of NDs-DMSO-ZS is smooth, indicating that the Zn^2+^ plating/stripping process is more uniform during the cycle process, which is conducive to the long-term cycle stability of the cell.

**Fig. 3:**
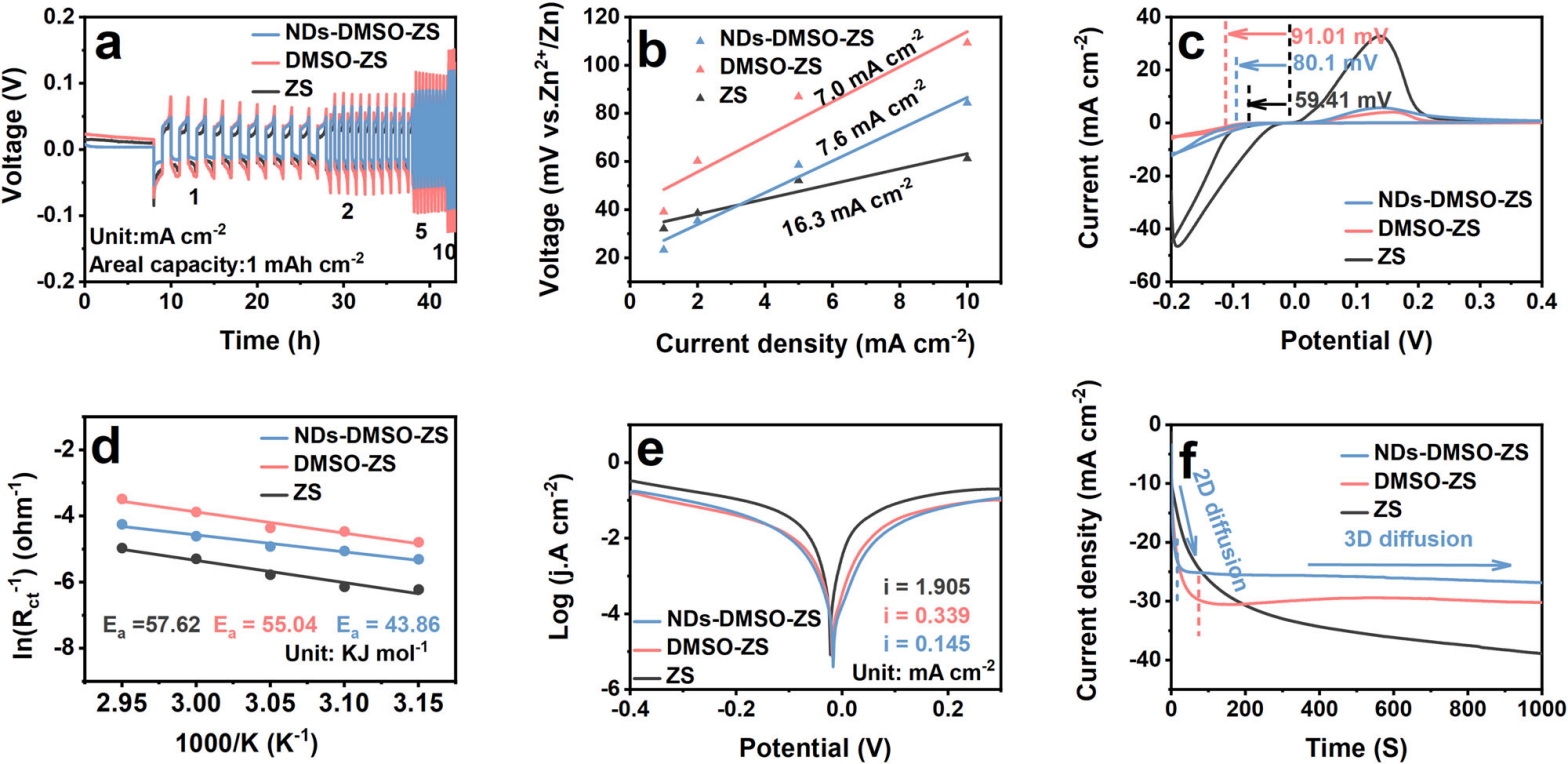
The electrochemical performance of Zn||Zn symmetric cells and Zn//Ti asymmetric cells in different electrolytes. **a** Rate performances of the Zn||Zn symmetric cells in different current densities with a fixed capacity of 1 mAh cm^−2^. **b** Exchange current density from curves in (**a**). **c** Cyclic voltammograms (CV) for Zn nucleation on bare Ti foil in different current densities under the scan rate of 1 mV s^−^^1^. **d** Arrhenius curves of ZS, DMSO-ZS, and NDs-DMSO-ZS. **e** Tafel test of the Zn||Zn symmetric cells in different electrolytes. **f** Chronoamperograms (CAs) at an overpotential of −150 mV.

To investigate the influence of NDs-DMSO on the Zn deposition kinetics, we subsequently performed electrochemical performance tests on different cells. As shown in Fig. 3a, the rate performance of Zn | |Zn symmetric cells with a fixed areal capacity of 1 mAh cm^−^^2^. Compared with the cells using ZS alone, the symmetric cells using DMSO-ZS exhibit higher overpotential. When DMSO participates in the Zn^2+^ solvation sheath, the hydrated Zn^2+^ exists in the form of [Zn(H_2_O)_m_(DMSO)_n_]^2+^, and the radius of Zn^2+^ solvation sheath increases, which may lead to the slower Zn^2+^ diffusion rate in the electrolyte, resulting in significantly increased overpotential. It has been reported that proper overpotential can suppress HER, but too high overpotential will affect Zn deposition kinetics^15,50^. However, the introduction of NDs slightly reduces the overpotential. The reason may be that NDs provide abundant active sites (-OH, -COOH, C = O) for Zn deposition, especially the surface -COOH groups that can strongly attract cationic Zn^2+^, increasing the concentration of Zn^2+^ at EEI, which is beneficial to reducing overpotential^51,52^. In addition, due to NDs can provide more zoophilic nucleation sites for the Zn^2+^ plating/stripping, which is also favorable for reaction kinetics^53^. For a more comprehensive assessment of the Zn deposition kinetics, the exchange current density is calculated by Eq. (2)^54^:

2 $${{\rm{i}}} \, \approx \, {i}_{0}\frac{F}{{RT}}\frac{\eta }{2}$$

where $i$ is the current density, ${i}_{0}$ is the exchange current density, $\eta$ is the total overpotential, $F$ is the Faraday constant, $R$ is the gas constant, and $T$ is the absolute temperature. As shown in Fig. 3b, DMSO is added to the electrolyte only, and the exchange current density value is the smallest (7.0 mA cm^−2^), indicating relatively slow Zn deposition kinetics. Simultaneously, NDs slightly increase the exchange current density (7.6 mA cm^−2^), which is conducive to improving the Zn deposition kinetics^53^. To study the initial stage of the Zn deposition behavior, the Zn//Ti asymmetric cells were characterized by cyclic voltammetry (CV). In the initial stages of Zn nucleation and growth, the nucleation overpotential (NOP) serves as a parameter to explain the extent of electrode polarization. Generally, the relationship between the critical Zn nucleus radius (${r}_{{crit}}$) and the NOP (${{\rm{\eta }}}$) is by Eq. (3)^55^:

3 $${r}_{{crit}}=2\frac{\gamma {V}_{m}}{F{{\rm{|}}}\eta {{\rm{|}}}}$$

Here, ${{\rm{\gamma }}}$ represents the surface energy at the electrode-electrolyte interface, ${V}_{m}$ is the Zn molar volume, and ${{\rm{F}}}$ is the Faraday’s constant. According to Eq. (3), ${r}_{{crit}}$ shows a clear inverse relationship with ${{\rm{\eta }}}$^56^. In the ZS, a low NOP (59.41 mV) indicates that the initial Zn nuclei are large, which is not beneficial to uniform Zn deposition, leading to the growth of Zn dendrites. In contrast, DMSO can promote Zn nucleus fineness, which is beneficial to achieving dense and uniform Zn deposition behavior (Fig. 3c)^57^. After adding NDs, NOP decreased slightly, which may be due to the regulation of HBs. Under the action of hydrogen bonding force, DMSO and H_2_O in [Zn(H_2_O)_m_(DMSO)_n_]^2+^ smoothly leave the solvation sheath, reducing energy consumption and lowering the Zn^2+^ nucleation barrier during the Zn^2+^ de-solvation process, increasing kinetics. Subsequently, we tested the charge-transfer resistance (R_ct_) of Zn | |Zn symmetric cells at different temperatures and calculated the de-solvation activation energy of different electrolytes by the Arrhenius equation. The R_ct_ at different temperatures (45-65 曟) can be obtained from the EIS plots (Fig. S4). Generally speaking, a lower E_a_ value is beneficial for promoting the de-solvation process of hydrated Zn^2+^^48^. As shown in Fig. 3d, the Zn | |Zn symmetric cells using NDs-DMSO-ZS have the smallest E_a_ value (43.86 KJ mol^−^^1^), indicating that the NDs-DMSO additive has a positive effect on the kinetics during Zn plating. This can be attributed to HBs, which significantly reduce the activation energy barrier of Zn^2+^ de-solvation under the action of hydrogen bonding force. DMSO and H_2_O in [Zn(H_2_O)_m_(DMSO)_n_]^2+^ smoothly leave the solvation sheath, reducing energy consumption and promoting the reduction of Zn^2+^ to Zn^58^.

Additionally, H_2_O in the Zn^2+^ solvation sheath can trigger various side reactions, including hydrogen evolution and corrosion. The linear sweep voltammetry (LSV) test results of Zn//Ti asymmetric cells indicate a shift towards the negative direction in the hydrogen evolution potential of NDs-DMSO-ZS (Fig. S5), which may be due to the surface groups (-COOH) increases the overpotential of H^+^ reduction, inhibiting HER^21,52^. As shown in Fig. 3e, the corrosion current densities for three different electrolytes were determined through Tafel fitting: NDs-DMSO-ZS (0.145 mA cm^−2^), DMSO-ZS (0.339 mA cm^−2^), and pure ZS (1.905 mA cm^−2^). The results show that the corrosion current density of NDs-DMSO-ZS is the smallest, meaning a lower corrosion reaction rate on its Zn anode surface. The lower corrosion reaction rate indicates that the Zn anode has good anti-corrosion, which helps to suppress the formation of Zn dendrites^59^. In particular, due to the various surface functional groups on NDs, they also interact with H_2_O through the HBs while interacting with DMSO. This interaction also reduces the quantity of active free H_2_O on the surface of the Zn anode, thereby enhancing anti-corrosion^21,22^. Finally, as shown in Fig. 3f, the working mechanism of regulating Zn deposition behavior is studied by chronoamperometry (CA) testing. At an overpotential of −150 mV, the current density of the cells using ZS exhibits a linear increasing trend throughout the entire testing period. The Zn^2+^ is influenced by the tip effect, leading to their preferential accumulation at the tips, ultimately evolving into Zn dendrites, indicating that Zn^2+^ undergoes uncontrolled diffusion along the two-dimensional (2D) direction. In the cells using DMSO-ZS and NDs-DMSO-ZS, their current densities instantaneously increase within the first 20 to 80 s of the process, indicating a rapid 2D diffusion process at the initiation of Zn deposition. Subsequently, the current densities tend to stabilize, suggesting that the Zn^2+^ diffusion has shifted to a three-dimensional (3D) phase. In general, the Zn nucleus can form rapidly in the initial stages of Zn nucleation, and the stability of Zn deposition kinetics will not be affected by the nanoparticles (NDs)^46,60^.

**Fig. 4:**
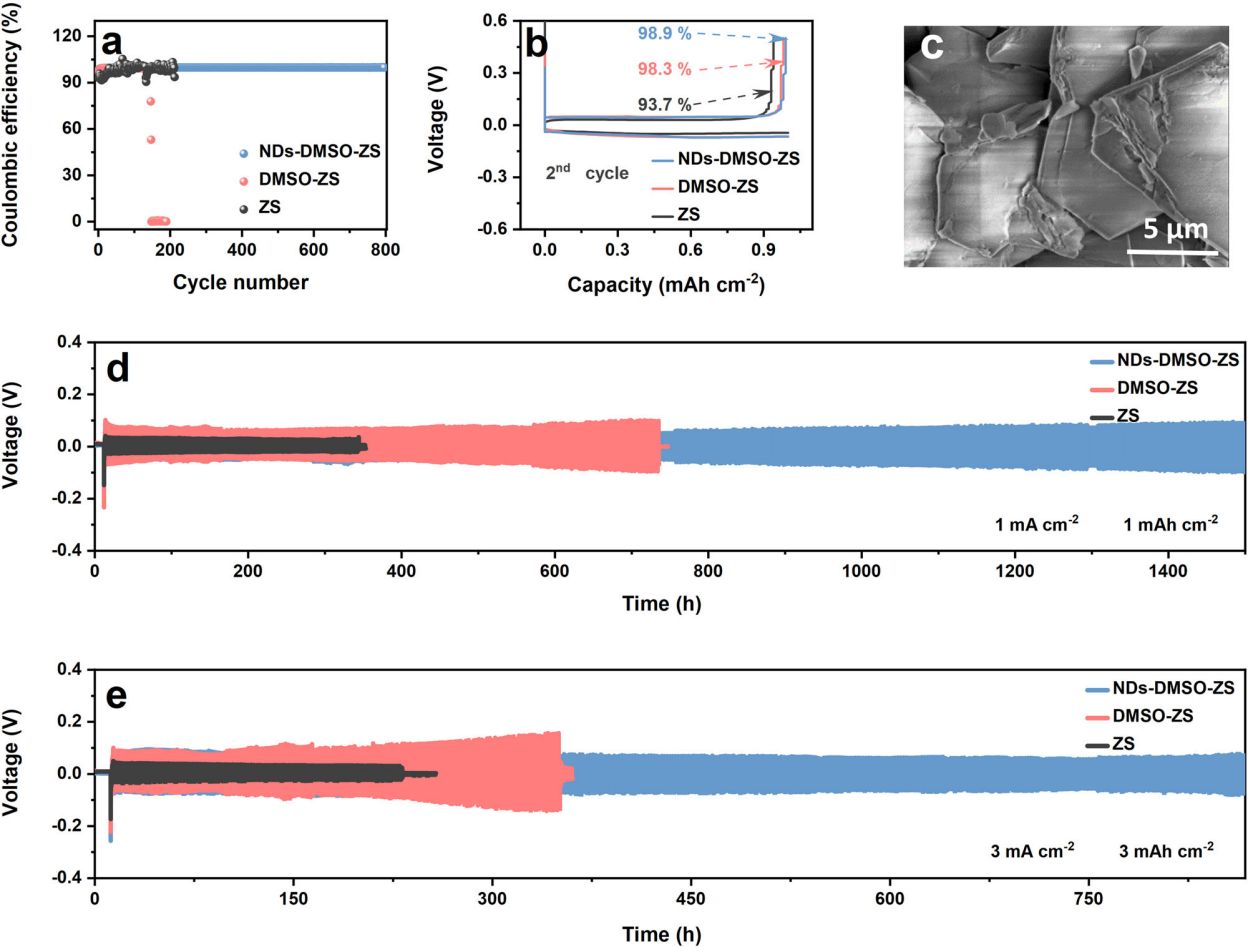
The performance of Zn//Cu asymmetric cells and Zn||Zn symmetric cells in different electrolytes. **a** Coulombic efficiency (CE) of the Zn//Cu asymmetric cells in different electrolytes. **b** Galvanostatic Charge-Discharge (GCD) profiles of the Zn//Cu asymmetric cells in different electrolytes. **c** SEM of the Zn anode after the cells failure at 3 mA cm^−2^ for 3 mAh cm^−2^ in NDs-DMSO-ZS. Cycling performance of Zn||Zn symmetric cells in different electrolytes at (**d**) 1 mA cm^−2^ for 1 mAh cm^−2^ and (**e**) 3 mA cm^−2^ for 3 mAh cm^−2^.

Next, we tested the CE of Zn//Cu asymmetric cells, as illustrated in Fig. 4a. The Zn//Cu asymmetric cells using DMSO-ZS and NDs-DMSO-ZS exhibit stable initial cycling, with no significant fluctuations observed. As the cycle progresses, the Zn//Cu asymmetric cells using DMSO-ZS maintain less than 200 cycles, and the CE curve indicates cell failure^59^. By contrast, the Zn//Cu asymmetric cells introducing NDs-DMSO provide more than 800 effective cycles, maintaining a high cycling stability (99.8%). Benefiting from the rich surface functional groups, the interaction between DMSO and NDs improves the Zn^2+^ diffusion rate; NDs also interact with H_2_O to enhance anti-corrosion. Therefore, NDs are very important for the long-term cycling stability of the cells. Figure 4b illustrates the initial CE of Zn//Cu asymmetric cells using ZS, DMSO-ZS, and NDs-DMSO-ZS, which are 93.7%, 98.3%, and 98.9%, respectively^6,15^. To further investigate the influence of NDs-DMSO on the long-term stability and reversibility of AZIBs, we conducted repeated charge-discharge tests on the Zn | |Zn symmetric cells. As shown in Fig. 4d, the Zn | |Zn symmetric cells using NDs-DMSO-ZS exhibit impressive cycling stability, surpassing 1500 hours at a current density of 1 mA cm^−2^. By contrast, the Zn | |Zn symmetric cells using ZS and DMSO-ZS can only sustain cycles for 342 hours and 733 hours, respectively. As expected, as the cycle progresses, the cells using DMSO-ZS exhibit significant electrode polarization, ultimately leading to cell failure. This may be due to the shorter chain lengths and smaller end steric hindrances of DMSO molecules, which facilitate the formation of dense structures and coordination interaction organic interface layer at EEI. The organic interface layer will slow down the reduction reaction kinetics of Zn^2+^, hinder the Zn^2+^ de-solvation process, and lead to intensified interface polarization^20,61–63^. In general, electrode polarization is more prominent at high current density, but the current density is increased to 3 mA cm^−2^ (corresponding to a surface capacity of 3 mAh cm^−2^), as shown in Fig. 4e. The Zn | |Zn symmetric cells using NDs-DMSO-ZS show cycling stability (800 h), which is an exciting result. After the cells failed, we disassembled them and characterized the morphology of the Zn anode surface (Fig. 4c, Fig. S6). In stark contrast, the Zn | |Zn symmetrical cells using NDs-DMSO-ZS exhibit a clear hexagonal structure growing outward on the Zn anode surface. As reported, outward growth deposition can prevent the formation of Zn dendrites, effectively prolonging the lifetime of the Zn^2+^ plating/stripping^64^. Under the synergistic effect of DMSO and NDs, Zn dendrites and other side reactions are effectively suppressed, reducing the effects caused by electrode polarization and promoting the long-term cycling stability of the cells^65–67^.

**Fig. 5:**
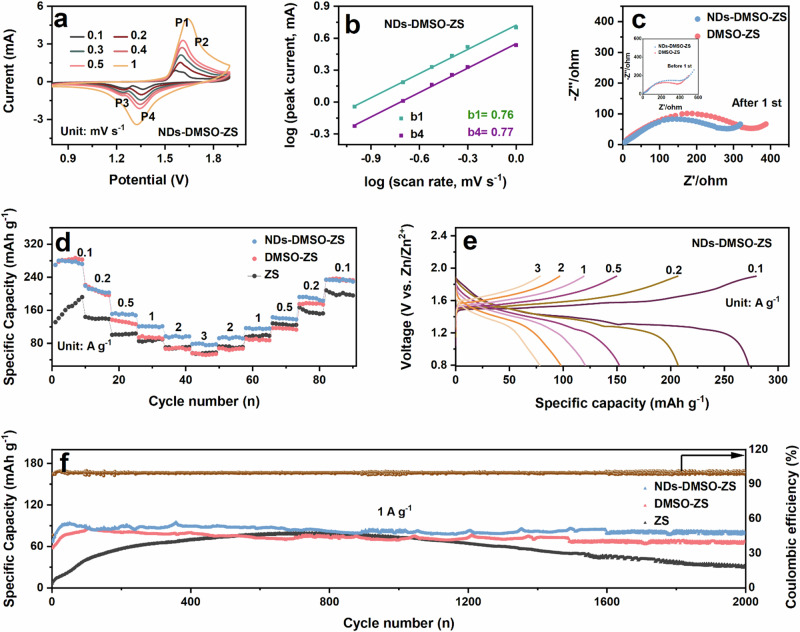
The electrochemical performance of Zn//MnO_2_ full cells in different electrolytes. **a** CV curves of the Zn//MnO_2_ full cells of NDs-DMSO-ZS with the scan rate from 0.1 to 1.0 mV s^−^^1^. **b** Log (i) vs log (v) plots of two peaks in CV curves. **c** EIS curves of the Zn//MnO_2_ full cells in different electrolytes. **d** Rate performance of the Zn//MnO_2_ full cells from 0.1 to 3 A g^−^^1^ in different electrolytes. **e** Charge-discharge profiles of rate performance in the NDs-DMSO-ZS. **f** Cyclic stabilities and efficiencies of the Zn//MnO_2_ full cells in different electrolytes at a current density of 1 A g^−^^1^.

To demonstrate the feasibility of the NDs-DMSO additive in a practical battery system, the Zn//MnO_2_ full cells were assembled using different electrolytes. As shown in Fig. 5a, cyclic voltammetry (CV) testing results indicate that the Zn//MnO_2_ full cells using NDs-DMSO-ZS demonstrate two noticeable redox peaks at a scanning rate of 0.1 mV s^−^^1^. The redox peaks gradually broaden as the scan rate increases from 0.1 mV s^−^^1^ to 1 mV s^−^^1^. Nevertheless, the overall shape of the CV curves remains consistent throughout this process. Assuming that the relationship between the current and the scan rate according to the following equation^68^:

4 $${{\rm{i}}}={{\rm{a}}}{\nu }^{b}$$

it can be rewritten as

5 $$\log \left({{\rm{i}}}\right)={{\rm{blog}}}\left({{\rm{\nu }}}\right)+\log \left({{\rm{a}}}\right)$$

where $i$ refers to current, $v$ stands for scan rate, and the corresponding adjustable parameters are $a$ and $b$. Through fitting $\log (i)$ versus $\log (v)$, the coefficient $b$ for peaks 1 and 4 can be determined based on the slope of the linear regression lines. The calculated $b$ values for peaks 1 and 4 are theoretically 0.76 and 0.77, respectively (Fig. 5b). When the parameter “$b$” exceeds 0.5, it indicates that the pseudocapacitive redox reaction plays a predominant role throughout the whole storage process, which determines the high rate performance of the cells^68,69^. Compared to the cells using NDs-DMSO-ZS, the others exhibit relatively small “$b$” values, indicating a lower contribution from their pseudocapacitive behavior(Fig. S7). Additionally, as shown in Fig. 5c, the charge transfer kinetics at EEI was studied by electrochemical impedance spectroscopy (EIS). The semicircle in the high-frequency region represents the interfacial charge transfer resistance (R_ct_), while the straight line in the low-frequency region represents the diffusion resistance^70^. The results show that the Zn//MnO_2_ full cells using NDs-DMSO-ZS exhibit larger R_ct_ before the first charge-discharge cycle (see insets for EIS graphs). It may be that NDs play a certain degree of particle obstruction in the electrolyte, resulting in a large impedance at EEI. After the first cycle, the R_ct_ of the Zn//MnO_2_ full cells using NDs-DMSO-ZS is smaller than the DMSO-ZS. This phenomenon reflects the vital function of NDs surface functional groups, which promote the diffusion and charge transfer of Zn^2+^ at EEI^37,38^.

In addition, we also performed rate performance tests on the Zn//MnO_2_ full cells using different electrolytes (Fig. 5d). The Zn//MnO_2_ full cells using NDs-DMSO-ZS exhibit excellent rate performance, exhibiting high reversible specific capacities of 272.3, 206.6, 152.1, 120.4, 97.7, and 78.1 mAh g^−^^1^ at current densities of 0.1, 0.2, 0.5, 1, 2, and 3 A g^−^^1^, respectively. It can be attributed to the fast reaction kinetics and remarkable Zn^2+^ plating/stripping behavior, which maintained excellent rate performance. In contrast, the reversible specific capacities of the Zn//MnO_2_ full cells using DMSO-ZS and ZS are relatively lower, showing notably poorer rate performance at different current densities^71^. Additionally, it is noteworthy that during the constant current charge-discharge process shown in Fig. 5e and Fig. S8, all Zn//MnO_2_ full cells display two distinct sloping charge-discharge plateau curves. These plateau curves correspond to the insertion and extraction of Zn^2+^, showing good agreement with the CV curve results and remaining consistent with previous research^8^. Finally, as shown in Fig. 5f, the Zn//MnO_2_ full cells using NDs-DMSO-ZS demonstrate a sustained reversible capacity of 80.8 mAh g^−^^1^ over more than 2000 cycles at a current density of 1 A g^−^^1^ (with a mass loading of 2.1 mg cm^−2^). The outstanding performance of the cells can be primarily attributed to the synergistic effect of DMSO and NDs, working together to inhibit the growth of Zn dendrites and suppress the occurrence of other side reactions. This study also highlights the substantial potential of NDs in the long-term cycling stability of AZIBs.

## Conclusion

In summary, this study optimizes Zn deposition behavior by introducing NDs-DMSO into the ZnSO_4_ electrolyte, and this paper also demonstrates the effectiveness of NDs-DMSO in suppressing the growth of Zn dendrites and other side reactions. The introduction of DMSO helps reduce side reactions triggered by active free H_2_O. However, as a strongly polar molecule, DMSO has a strong adsorption capacity for Zn, which is not beneficial to the reaction kinetics and increases electrode polarization. Due to the HB-donor groups (-OH, -COOH)) of NDs and the HB-acceptor groups (S = O), NDs can interact with DMSO by the strong intermolecular HBs to reduce electrode polarization. Specifically, the strong intermolecular HBs decrease unnecessary energy consumption that DMSO causes during the de-solvation process, improve reaction kinetics, and reduce electrode polarization. The test results indicate that the cells using NDs-DMSO-ZS exhibit excellent cycling stability and high CE. This study provides a feasible and efficient way to improve the performance of AZIBs by optimizing the electrolyte. At the same time, we hope to provide a reference for the future design of commercially viable high-performance AZIBs.

## Methods

### Electrolyte and electrode preparation

The bare 2 M ZnSO_4_ electrolyte (ZS) was prepared by dissolving 11.502 g ZnSO_4_·7H_2_O into 20 mL deionized water under vigorous magnetic stirring for 30 min. The DMSO solution was directly added to the pre-prepared ZS (volume ratio: 1:5) to form the DMSO-ZS. The 5 mg NDs were added to 5 mL DMSO solution and subjected to ultrasonication for 1 hour to create an NDs-DMSO mixed solution, which served as an electrolyte additive. Subsequently, the NDs-DMSO mixed solution was added to the pre-prepared ZS (volume ratio: 1:5) to form the NDs-DMSO-ZS. In addition, 0.2 M MnSO_4_·H_2_O was added to the above electrolyte as the electrolyte of the Zn//MnO_2_ cells. The MnO_2_ cathode was composed of 70 wt.% commercial powder of active material MnO_2_, 20 wt.% super P, and 10 wt.% PVDF with N-methyl-2-pyrrolidine as the solvent. Hydrophilic type carbon paper served as the current collector. The slurry was uniformly coated on carbon paper and then dried in a vacuum at 80 °C for 12 h. The carbon paper was cut into a small disc-shaped wafer (12 mm in diameter) with a mass loading of 2.1 mg cm^−2^ used as the cathode.

### Material characterizations

The elements, morphologies, and crystalline structures of the electrodes and electrolytes were examined through scanning electron microscopy (SEM, S-4800, Hitachi Limited) equipped with energy dispersive spectroscopy (EDS) profiles and transmission electron microscopy (TEM, JEM-2100F, JEOL). After the cycling processes, the Zn electrodes were removed from the cells and the surface of the Zn electrodes was cleaned with a large amount of deionized water. Finally, the Zn electrodes were ultrasonic-treated in alcohol for 30 min, and the supernatant was collected for the preparation of TEM samples.

### Electrochemical measurements

The Zn | |Zn cells, Zn//Cu cells, and Zn//MnO_2_ cells were assembled to evaluate the electrochemical performances. They were based on a CR2025 coin cell in Land Battery Measurement System (CT2001 1 A), with a cut-off voltage of 0.8–1.9 V. The amounts of the electrolyte for each cell were controlled at 150 μL. The thickness of Zn electrodes is 70 μm. The electrochemical impedance spectroscopy (EIS) was measured over a frequency ranging from 100 kHz to 0.01 Hz with an amplitude of 5 mV. The cyclic voltammetry (CV) measurement was carried out at different scan rates between 0.8 V and 1.9 V. The Chronoamperometry (CA) of the Zn | |Zn symmetric cells at an overpotential of −150 mV, linear polarization curves at 5 mV s^−^^1^ based on the Zn | |Zn symmetric cells, and LSV curves based on Zn//Ti half cells at 5 mV s^−^^1^ and Zn//MnO_2_ cells at 1 mV s^−^^1^. They were measured on a CHI660E electrochemical workstation (ChenHua, Shanghai).

### Supplementary information

Supplementary Information

## Data Availability

The data that support the findings of this study are available from the corresponding author upon reasonable request.
