# Supplementary material for: A dual-functional electrolyte additive displaying hydrogen bond fusion enables highly reversible aqueous zinc ion batteries
Source: Commun Chem. 2024 Aug 8;7:173. doi: 10.1038/s42004-024-01259-3 (PMC11310298; doi:10.1038/s42004-024-01259-3)
Supplement: Supplementary file 1 — Supplementary Information [file 42004_2024_1259_MOESM1_ESM.pdf]

Supplemental Information

The dual-functional electrolyte additive with hydrogen bond fusion for building highly reversible aqueous zinc ion batteries

Qiuxia Zhang,[1] Xuan Gao,*[2,3] Kejiang Liu,[1] Nan Gao,[1] Shaoheng Cheng,[1] Yuhang Dai,[3] Haobo Dong,[4] Junsong Liu,*[1] Guanjie He,*[2] Hongdong Li*[1]

[1]State Key Laboratory of Superhard Materials, College of Physics, Jilin University, Jilin, Changchun, 130012 (P. R. China)

[2]Christopher Ingold Laboratory, Department of Chemistry, University College London, 20 Gordon Street, London, WC1H 0AJ (UK)

[3]Department of Engineering Science, University of Oxford, 17 Parks Road, Oxford, OX1 3PJ (UK)

[4]School of Future Technology, South China University of Technology, 381 Wushan Road, Tianhe District, Guangzhou, 510641 (P. R. China)

Experimental section:

1. Electrolyte and electrode preparation

The bare 2 M $ZnSO_4$ electrolyte (ZS) was prepared by dissolving 11.502 g $ZnSO_4·7H_2O$ into 20 mL deionized water under vigorous magnetic stirring for 30 min. The DMSO solution was directly added to the pre-prepared ZS (volume ratio: 1:5) to form the DMSO-ZS. The 5 mg NDs were added to 5 ml DMSO solution and subjected to ultrasonication for 1 hour to create an NDs-DMSO mixed solution, which served as an electrolyte additive. Subsequently, the NDs-DMSO mixed solution was added to the pre-prepared ZS (volume ratio: 1:5) to form the NDs-DMSO-ZS. In addition, 0.2 M $MnSO_4·H_2O$ was added to the above electrolyte as the electrolyte of the Zn//MnO_2 cells. The MnO_2 cathode was composed of 70 wt.% commercial powder of active material MnO_2, 20 wt.% super P, and 10 wt.% PVDF with N-methyl-2-pyrrolidine as the solvent. Hydrophilic type carbon paper served as the current collector. The slurry

was uniformly coated on carbon paper and then dried in a vacuum at 80 °C for 12 h. The carbon paper was cut into a small disc-shaped wafer (12 mm in diameter) with a mass loading of 2.1 mg cm^{-2} used as the cathode.

2. Material characterizations

The elements, morphologies, and crystalline structures of the electrodes and electrolytes were examined through scanning electron microscopy (SEM, S-4800, Hitachi Limited) equipped with energy dispersive spectroscopy (EDS) profiles and transmission electron microscopy (TEM, JEM-2100F, JEOL). After the cycling processes, the Zn electrodes were removed from the cells and the surface of the Zn electrodes was cleaned with a large amount of deionized water. Finally, the Zn electrodes were ultrasonic-treated in alcohol for 30 min, and the supernatant was collected for the preparation of TEM samples.

3. Electrochemical measurements

The Zn||Zn cells, Zn//Cu cells, and Zn//MnO_2 cells were assembled to evaluate the electrochemical performances. They were based on a CR2025 coin cell in Land Battery Measurement System (CT2001 1 A), with a cut-off voltage of 0.8–1.9 V. The amounts of the electrolyte for each cell were controlled at 150 μl. The thickness of Zn electrodes is 70 μm. The electrochemical impedance spectroscopy (EIS) was measured over a frequency ranging from 100 kHz to 0.01 Hz with an amplitude of 5 mV. The cyclic voltammetry (CV) measurement was carried out at different scan rates between 0.8 V and 1.9 V. The Chronoamperometry (CA) of the Zn||Zn symmetric cells at an overpotential of -150 mV, linear polarization curves at 5 mV s^{-1} based on the Zn||Zn symmetric cells, and LSV curves based on Zn//Ti half cells at 5 mV s^{-1} and Zn//MnO_2 cells at 1 mV s^{-1}. They were measured on a CHI660E electrochemical

workstation (ChenHua, Shanghai).

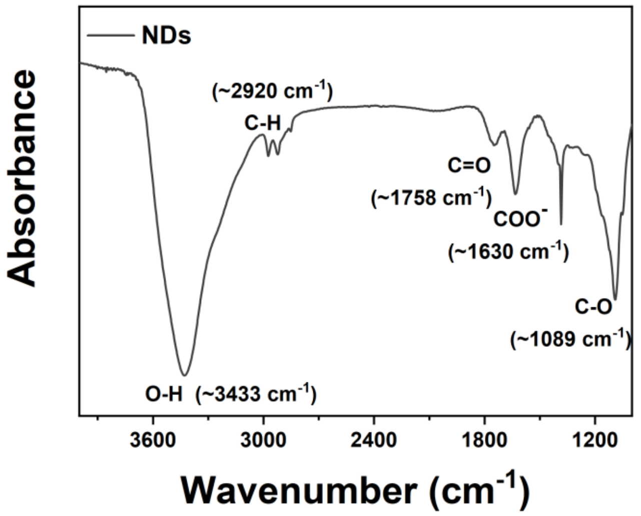

Fig. S1 FTIR spectra of the NDs

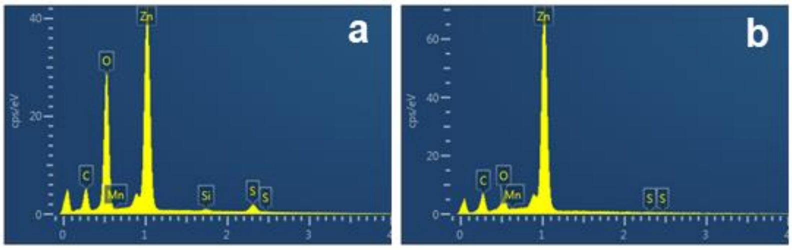

Fig. S2 EDS mapping of the Zn anode after 2 hours cycling at 10 mA cm^{-2} for 10 mAh cm^{-2} (a) ZS and (b) NDs-DMSO-ZS.

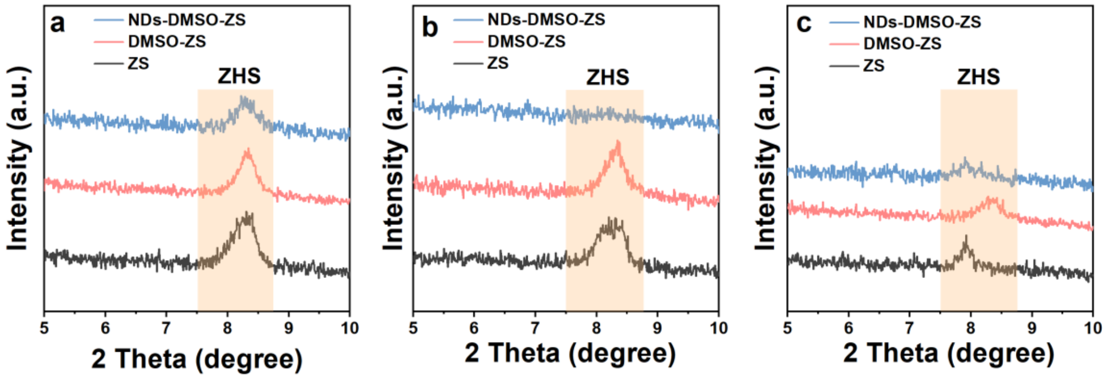

Fig. S3 Ex Situ XRD spectra of MnO_2 cathodes with different electrolytes after cycling at a current density of 2 A g^{-1} at different potential states (a) the first discharge (0.8 V), (b) the first charge (1.9 V),

and (c) the second discharge (1.6 V).

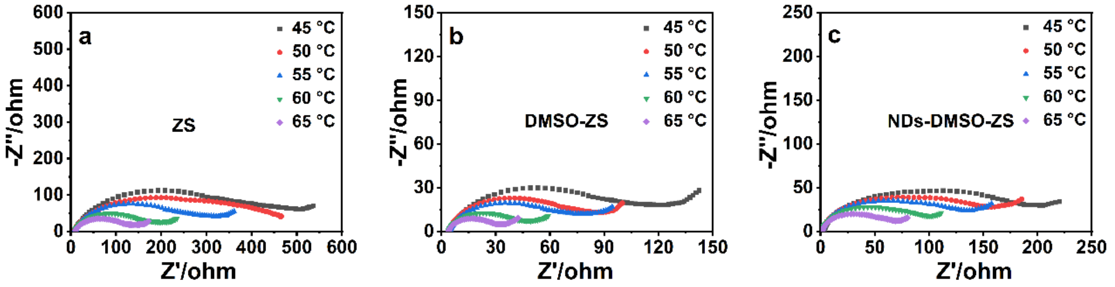

Fig. S4 EIS profiles of the symmetric cells using (a) ZS, (b) DMSO-ZS, and (c) NDs-DMSO-ZS under different temperatures.

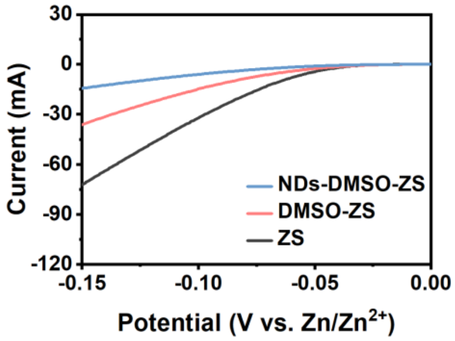

Fig. S5 LSV test of the Zn//Ti asymmetric cells in different electrolytes.

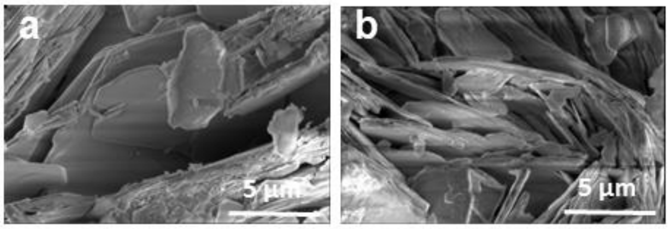

Fig. S6 SEM of the Zn anode after the cells failure at 3 mA cm^{-2} for 3 mAh cm^{-2} in (a) ZS and (b) DMSO-ZS.

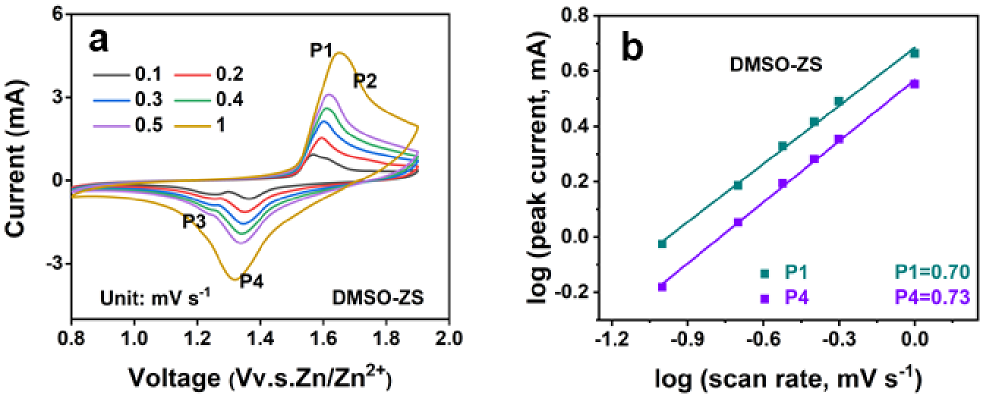

Fig. S7 (a) CV curves of the Zn//MnO_2 cells of DMSO-ZS with the scan rate from 0.1 to 1.0 mV s^{-1}. (b) Log (i) vs log (v) plots of two peaks in CV curves.

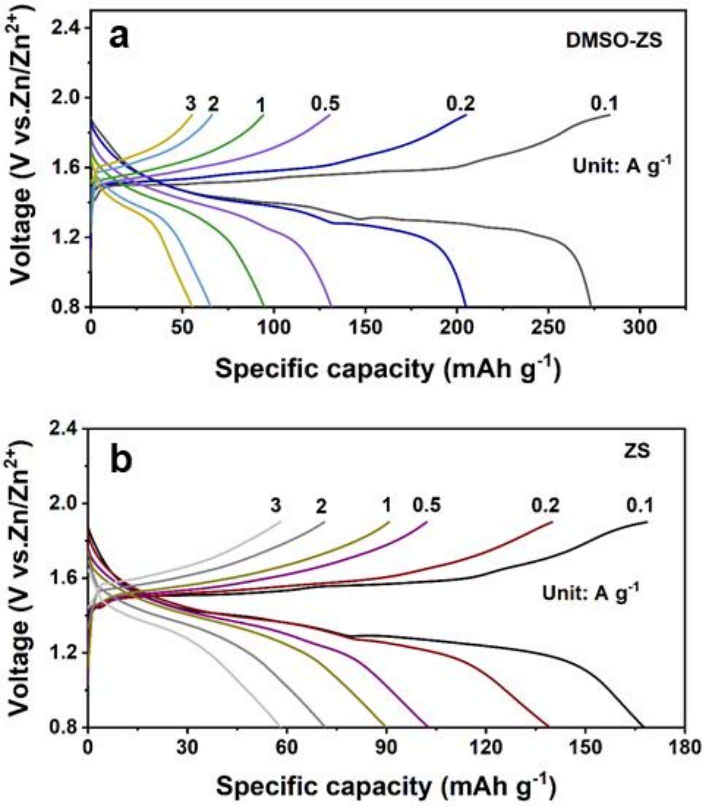

Fig. S8 Charge/discharge profiles of rate performance of the Zn//MnO_2 cells of (a) DMSO-ZS and (b) ZS.

NO	VALUE	TMP
ZS	43.2 mS/cm	24.6 ℃
DMSO-ZS	20.2 mS/cm	23.9 ℃
NDs-DMSO-ZS	19.84 mS/cm	24.9 ℃

Fig. S9 The ionic conductivity of the different electrolytes.

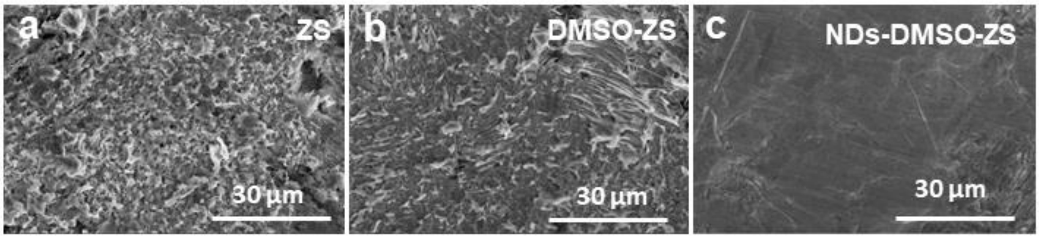

Fig. S10 The SEM images of the Zn anode surface after 2 hours cycling at 10 mA cm^{-2} for 10 mAh cm^{-2} in (a) ZS, (b)DMSO-ZS, and (c) NDs-DMSO-ZS.

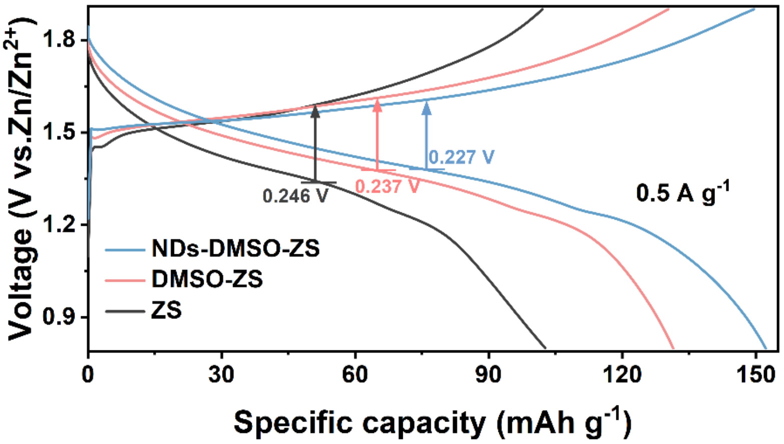

Fig. S11 Charge/discharge profiles of the Zn//MnO_2 full cells in different electrolytes at the current density of 0.5 A g^{-1}.
